# Can We Use Grip Strength to Predict Other Types of Hand Exertions? An Example of Manufacturing Industry Workers

**DOI:** 10.3390/ijerph18030856

**Published:** 2021-01-20

**Authors:** Victor Ei-Wen Lo, Yi-Chen Chiu, Hsin-Hung Tu

**Affiliations:** 1 Department of Occupational Safety and Health, China Medical University, Taichung City 40604, Taiwan; u101014303@cmu.edu.tw; 2 Department of Computer-Aided Industrial Design, Overseas Chinese University, Taichung City 40721, Taiwan; josephtu@ocu.edu.tw

**Keywords:** strength, prediction model, grip, lateral pinch, palmar pinch, thumb press, ball of thumb, manufacturing

## Abstract

Background: There are different types of hand motions in people’s daily lives and working environments. However, testing duration increases as the types of hand motions increase to build a normative database. Long testing duration decreases the motivation of study participants. The purpose of this study is to propose models to predict pinch and press strength using grip strength. Methods: One hundred ninety-eight healthy volunteers were recruited from the manufacturing industries in Central Taiwan. The five types of hand motions were grip, lateral pinch, palmar pinch, thumb press, and ball of thumb press. Stepwise multiple linear regression was used to explore the relationship between force type, gender, height, weight, age, and muscle strength. Results: The prediction models developed according to the variable of the strength of the opposite hand are good for explaining variance (76.9–93.1%). Gender is the key demographic variable in the predicting models. Grip strength is not a good predictor of palmar pinch (adjusted-*R*^2^: 0.572–0.609), nor of thumb press and ball of thumb (adjusted-*R*^2^: 0.279–0.443). Conclusions: We recommend measuring the palmar pinch and ball of thumb strength and using them to predict the other two hand motions for convenience and time saving.

## 1. Introduction

Handgrip strength (HGS) data have been widely used in many fields. The measurement of grip strength is recommended by the World Health Organization (WHO) for the International Classification of functioning, disability, and health (ICFDH) [1]. Furthermore, physicians and researchers on aging have diagnosed some sarcopenia cases by grip strength. In addition, epidemiologists and physicians in public health and preventative medicine use the value of grip strength to predict the mortality of the elderly population [2,3,4,5,6,7]. Physical therapists and scientists determine the effectiveness of rehabilitation by measuring grip strength [8,9]. Handgrip strength tests were also used to determine the effects of interventions on strength levels in young athletes [10]. Furthermore, ergonomists, industrial engineers/designers, and industrial hygienists design and select appropriate hand tools and products to ensure the safety of manual tasks based on grip strength. Therefore, discovering the norm of grip strength for the general population has been done previously by researchers, physicians, physical therapists, and ergonomists [3,4,6,7,11,12,13,14,15,16,17,18,19,20,21,22,23,24,25,26,27,28,29,30,31]. In addition, there are two novel studies that measure and mention handgrip strength in children and haemodialysis patients [32,33].

Apart from the grip strength, there are other types of hand exertions existing in our daily lives or working environments [34,35,36,37,38]. For example, Shoorlemmer and Kanis showed that there were 11 types of hand exertions when interacting with controls, e.g., pinching, pressing, rotating, etc. [37]. Shaub et al. (2015) conducted a project and showed the eight types of most frequently used hand exertions in car and truck assembly plants in EU countries [36,39]. Furthermore, measurement of pinch strength has been done previously [15,16,18,19,20,24,26,27,28,40]. However, there were few studies measuring the thumb press, palm press, ball of thumb, or index finger press [29,39].

To build normative data on hand exertions, standard-testing procedures should be followed. Chaffin (1975) proposed an ergonomic guide on how to measure and report human strength [41]. The American Society of Hand Therapists (ASHT) proposed recommendations for the procedures and postures used to measure grip strength and three types of pinch strength [42]. Both guides recommend that measurements be taken three times on each hand to get valid and reliable data. Chaffin (1975) also recommended that experimenters should provide at least 2 min between testing trials to avoid muscle fatigue, if the total testing trials exceed 15 trials in one session [41]. In addition, a possible novel approach for measuring grip strength could be through smartphones or similar novel technologies since mobile phones are popular ways for measuring strength in different populations [43,44,45]. Moreover, in the application of normative data for evaluations and treatments, there are some demographic and anthropometric factors that should be considered. Demographic factors include age, gender, race, occupation, hand dominance, socioeconomic situation, nutrition, lifestyle, etc. Anthropometric variables include height, weight, body mass index, hand width, hand length, waist circumference, etc. It is hard to recruit study participants while measuring all the anthropometric variables described above because of the time constraints. Therefore, researchers attempted to propose models by applying these demographic and anthropometric variables to predict the grip strength and pinch strength more easily and accurately [11,13,14,15,16,17,18,19,20,21,22,23,24,46,47,48,49,50,51].

To follow the standard procedures of hand strength tests proposed by previous studies either using traditional measures or advanced technology, has a long testing duration. The testing duration increases as the number of trials of hand exertions to be measured increases. A long testing duration not only decreases the motivation of study participants but also results in localized muscle fatigue, which may affect the results. For example, Schaub et al. (2015) measured eight different types of finger‒hand forces used by the workers at several major European vehicle and trunk manufacturing companies [39]. Lo et al. (2019) also reported five types of hand exertions used by Taiwanese workers in manufacturing industries [29]. The test duration for one session was 1 h or longer. The numbers of hand exertions were 15 or more. The best solution to solve the problem of long testing periods is to divide them into two sessions. However, this may create another problem in that study participants may withdraw from the study. Another method is to propose a prediction model, considering not only demographic and anthropometric variables, but also the types of hand exertions. Previous studies focused on either grip strength or pinch strength [11,12,13,14,15,16,17,18,19,20,21,22,23,24,46,47,48,49,52]. Sung et al. (2015) developed grip and key pinch strength prediction models using the regression method and artificial neural networks (ANN) and found that there were no significant differences between the two models [16]. To the best of our knowledge, there are no studies investigating the relationship between grip strength, pinch strength, and press strength of finger‒hand exertions. Therefore, the purpose of this study is to propose models to predict different types of hand strength using grip strength.

## 2. Materials and Methods

### 2.1. Study Participants

A convenient sample of 198 healthy volunteers recruited from the manufacturing industries in Central Taiwan participated in this study. The sampling strategy and recruitment procedures are detailed elsewhere [29]. All volunteers were free of any diagnosed disorders, diseases or pain in the upper extremities in the past six months. In addition, they had no known diseases that may affect hand strength, such as rheumatoid arthritis or heart disease. When the volunteers sent emails or called for participation, the experimenter checked for those exclusion criteria and informed these volunteers that they could not drink caffeine or tea, and perform strenuous work one day before the study date if they were qualified to participate the study. These volunteers were distributed into five age groups of 20–25, 25–34, 35–44, 45–54, and 55–64 years with 32, 48, 48, 38, and 32 volunteers, respectively. All study volunteers received information regarding the study purposes, procedures, and data security process and signed the informed consent forms, approved by the Research Ethics Committee at China Medical University and Hospital (CMUH106-REC2-156), before participating in the study. There was no sex difference in terms of age (*p* = 0.974). The mean age was 39.1 years. There were significant sex differences in terms of height, weight, body mass index (BMI), and handspan. Detailed demographic and anthropometric information is given in Table 1.

### 2.2. Types of Hand Motions

**Figure 1:**
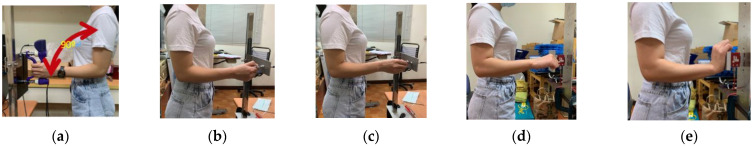
Five types of hand strength measured in this study: (**a**) power grip; (**b**) lateral pinch; (**c**) palmar pinch; (**d**) thumb press; and (**e**) ball of thumb press.

There are five different types of hand motions that are frequently used in the automotive and home appliance facilities in the manufacturing industry [39]. The five types of hand motions are power grip, lateral pinch, palmar pinch (3-jaw chuck pinch), thumb press, and ball of thumb press. The tests of these hand motions followed the instructions of Shaub et al. (2015) and Mathiowetz et al. (1984) [36,53], and are shown in Figure 1a–e, respectively. The posture of the shoulders and upper limbs for the power grip and two types of pinch exertions followed the guidelines recommended by the American Society of Hand Therapists (i.e., a standing position, instead of a sitting position). Volunteers stood upright and relaxed their shoulders. Their elbows flexed at 90° and the forearms were in a neutral position. For the two pressing tasks, volunteers’ elbows flexed at 90°, the forearms pronated at 90°, and the wrists were in a neutral position for the thumb press (Figure 1d) or flexed at 90° for the ball of thumb press (Figure 1e).

### 2.3. Equipment

A standardized digital handgrip dynamometer (G200, Biometrics, Ltd., Ynysddu, UK) was used to measure the power grip strength. The grip span ranged from 3.4 to 8.6 cm at 1.3 cm increments and the experimenter set the grip span based on the results of the study conducted by Ruiz et al. (2006) [54]. A digital pinchmeter (P200, Biometrics, Ltd., Ynysddu, UK) was used to measure lateral and palmar pinch strength. Both the grip dynamometer and pinchmeter were connected to a 16-channel BIOPCA MP 150 system (BIOPAC System, Inc., Goleta, CA, USA) via a general-purpose transducer amplifier (DA 100C, BIOPAC System, Inc., Goleta, CA, USA) for data acquisition and analysis. The sampling rate was 1000 Hz.

**Figure 2:**
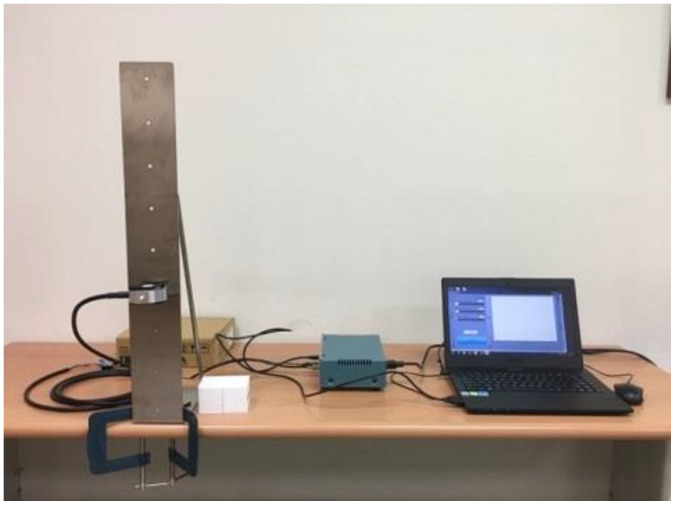
The setting of equipment for the measure of press strength.

Customized devices were designed to measure the strength of two types of press, the thumb press and ball of thumb (Figure 1d,e). A square of Teflon was attached to a S-Beam load cell (LSB302, Futek Advanced Sensor Technology, Inc., Irvine, CA, USA) and secured to a piece of L-shape stainless steel (20 cm Length × 10 cm width × 70 cm height) with a screw. There were 8 holes in the L-shape stainless steel starting from 3 cm above the base with a distance of 8 cm in between in order to adjust the height of the load cell (Figure 2). Signals were sent to a customized four-channel data acquisition box (NI DAQ USB-6002, National Instruments, Austin, TX, USA) and software written in LabVIEW (National Instruments, Austin, TX, USA) recorded the signals at a sampling rate of 100 Hz. Before the experiment started each day, the experimenters used the standard weights of 2 kg and 10 kg to calibrate the grip dynamometer, the pinchmeter, and load cells.

A questionnaire was designed to record demographic and anthropometric information, including participants’ name, age, gender, medical history (checking for the exclusion criteria), health conditions (pain or soreness), hand dominance, job information, height, weight, and handspan. A Martin-type anthropometer was used to measure the participants’ handspan, defined as the maximum distance from the tip of the thumb to the tip of the small finger with the hand open as wide as possible.

### 2.4. Procedures

When volunteers arrived, the experimenter asked questions related to demographic information, medical history, and health conditions, and then explained the instructions and procedures to the participant. If the volunteers did not have any questions, they signed the informed consent, and then started the experiment. Volunteers were instructed to exert their maximum force within 1–2 s in a standing position and maintained the force for at least 3 s until the experimenter said to stop [36]. Each type of hand motion was repeated three times. The mean of the 3 s data points represented the maximum strength of a specific trial. The average of three strength scores was used as the maximum strength for a specific type of hand motion. After the volunteers completed the three trials of a specific hand motion, the experimenter calculated the coefficient of variation (CV) to ensure the data quality (Equation (1)). If the CV was greater than 10%, the participant did it again until the CV was less than 10%. A minimum rest period of 3 min was provided between two repetitions. It took about 1 h to complete the experiment. For detailed information, please refer to our previous article [26].

(1) CV = σμ ×100%

where:

σ—the standard deviation

μ—the mean.

### 2.5. Statistical Analysis

The mean, standard deviation, and/or percent was used to present the demographic and strength data. Repeated measures ANOVA were used to determine the main effects and their interactions with age and type of hand motions. Bonferroni correction was used for post hoc analysis. Pearson correlation was conducted to determine the linear correlation between the variables. A *p*-value < 0.05 was used to determine statistical significance.

Regression analysis was used to explore the multivariate relationship of force pattern, gender, height, weight, and age to find a predictive model of muscle strength. In the regression equation, gender and age were the strongest related factors. We used the linearity, second-order interaction, and reverse curve of the dependent variable to calculate the curve fit of the two variables, and find the best one of each single factor that explained the maximum variance. We used XLSTAT software to perform the correlation of the second-order interaction items. Next, we combined all the single best fits into the stepwise multiple regression method to find the largest explanatory variance. Entry and removal probabilities were set at 0.05 and 0.10, respectively. The SPSS Chinese version 22.0 (IBM Corporation, Armonk, NY, USA) was used for statistical analyses.

## 3. Results

### 3.1. Sex, Types of Hand Motions, and Age: Effects on Hand Strength

**Figure 3:**
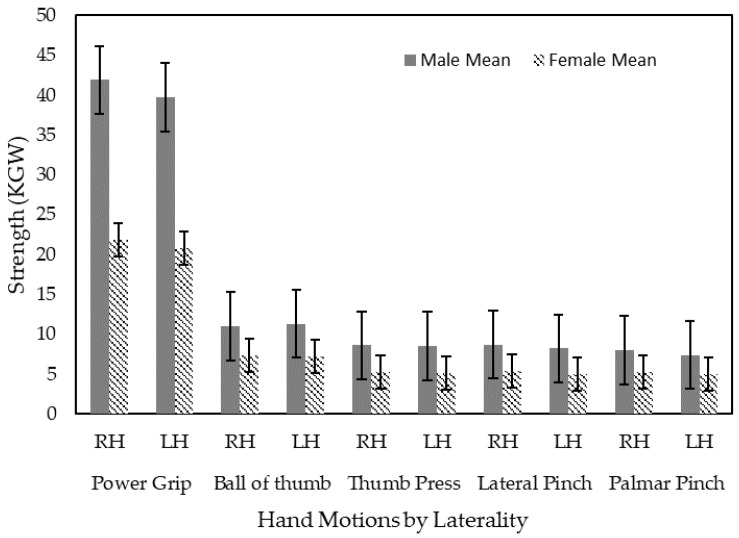
Hand strength by sex, hand laterality, and different types of hand motions.

It is not surprising that the strength of females was significantly lower than that of males, and rangin from 52.0% to 68% among the five types of hand motions (all *p* < 0.001) (Figure 3). Therefore, we performed the analysis by gender as follows.

**Figure 4:**
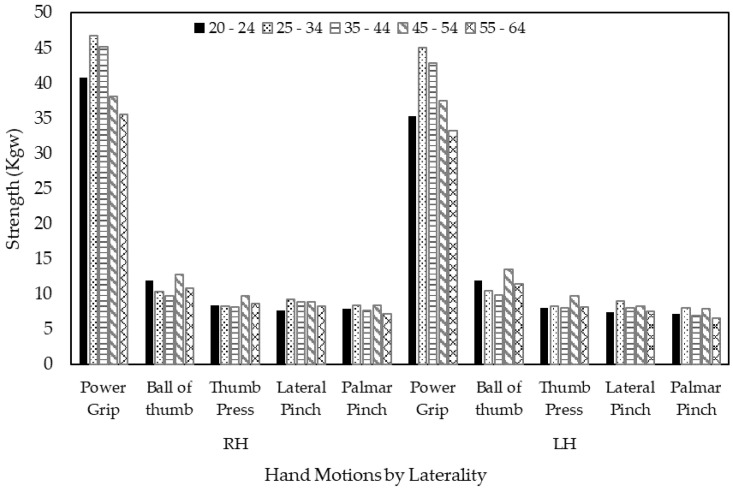
Age effects on hand strength by type of hand motion and laterality for male volunteers.

**Figure 5:**
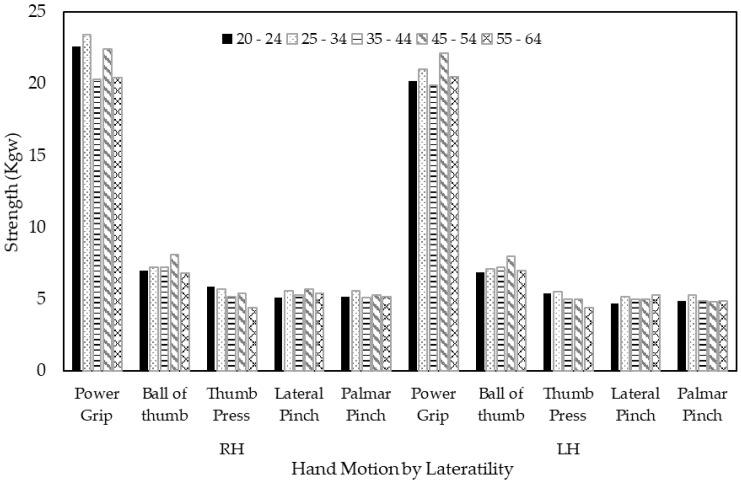
Age effects on hand strength by type of hand motion and laterality for female volunteers.

The repeated measures ANOVA results revealed that the type of hand motion had the main effect on the strength of both hands (F = 1078.381; df = 2.005, 188.461; *p* < 0.001 for right and F = 1094.502; df = 2.060, 193.595; *p* < 0.001 for left hands) for male volunteers (Figure 4). For female volunteers, likewise, the type of hand motion had the main effect on the strength of both hands (F = 830.198; df = 2.120, 199.298; *p* < 0.001 for right hands and F = 923.448; df = 2.045, 192.266; *p* < 0.001 for left hands) (Figure 5). Power grip strength was significantly greater than the other types of motion for both hands (*p* < 0.001). Meanwhile, there was no age effect on either hand for males or females. However, there were significant interactions between types of hand motions and age for both hands among males (*p* = 0.006 for right hand and *p* = 0.003 for left hand), but no such interaction effects were observed among females.

### 3.2. General Linear Models Using Demographic Variables

Prediction models of the types of hand motions for both hands based on the demographic information (for example, gender, age, height, and weight) are shown in Table 2. The demographic variables of gender and weight can be used to predict the maximum grip strength of the left hand (adjusted-*R*^2^ = 0.688). This is the model with the best explanatory power among the prediction models of demographic variables. The explanatory power for the grip strength of the right hand is 0.64 with the predictor of gender only. The third model with high explanatory power is the lateral pinch force (adjusted-*R*^2^ = 0.617), again based on gender only. The explained variance was 58.4% for the predicted model of the lateral pinch on the left hand with gender and weight. The explained variances for all other predicted models, e.g., palmar pinch, ball of thumb, and thumb press, based on the demographic variables, were less than 50%.

### 3.3. General Linear Model Using Strength of Opposite Hand and Demographic Variables

When the data for the left or right hand of five kinds of motion are known, the prediction of the strength of the other hand can be predicted by the known strength in combination with demographic variables, for example, gender, age, height, and weight. The combinations of these predictors account for approximately 75.6–93.2% of the variance and the prediction models with the adjusted-*R*^2^ are shown in Table 3. It is not surprising that the variances that can be explained were much better than the prediction models developed using only demographic variables. The adjusted-*R*^2^ values of the predicted models of grip strength, ball of thumb, and thumb press are all above 0.90 when including the variable of the strength of the opposite hand. The best model is for the ball of the thumb on the right hand, with an explained variance of 93.2%.

The variables of the predicted models for both lateral pinch strength and palmar pinch strength are the strength of the opposite hand and gender. However, the models can explain less than 90% of the variance. The adjusted-*R*^2^ for the lateral pinch strength on the right hand and left hand are 0.831 and 0.845, respectively. The model with the lowest adjusted-*R*^2^ predicted the palmar strength of the left hand using the measured strength of palmar pinch on the right hand and gender, with a value of 0.756.

Although the prediction models developed from the variable of the strength of the opposite hand explain the variance reasonably well, experimenters still need to measure all five types of hand exertions, which is time-consuming. Next, we would like to find the best prediction models, including the interaction terms. This means that experimenters would not need to measure all types of hand exertions and could shorten the experiment duration.

### 3.4. Prediction Models of the Hand Strength by Other Strength of Hand Motions, Demographic Variables, and Their Interactions

Since we measured five types of hand strength on both hands, we would like to propose stepwise linear models by laterality to predict strength using the types of hand motions, demographic variables, and the interaction of the demographic variables. The results are shown in Table 4. Among the five predictive models, gender and height are the most powerful predictors, regardless of handedness. In the models for the right hand, palmar pinch strength is the best predictor of grip strength and lateral pinch strength, in combination with other demographic variables. The overall adjusted-*R*^2^ was 0.79 and 0.777, respectively (*p* < 0.001). The same results can also be applied on the left hand and the overall adjusted-*R*^2^ values are 0.769 and 0.842 for grip strength and lateral pinch strength, respectively. However, the palmar pinch strength cannot be used to predict the two types of press strength for both hands. Measuring the palmar pinch strength and ball of thumb strength is recommended if the experiment has time constraints.

### 3.5. Prediction Models of Hand Strength by Grip Strength, Demographic Variables, and Interactions of Demographic Variables

Grip strength is the most frequent measurement used to evaluate the effectiveness of rehabilitation, predict mortality, and design tools and equipment. Furthermore, to reduce the duration of experiments and avoid having a large number of trials, which may result in muscle fatigue, we also evaluated the models using the measured grip strength with other demographic variables to predict another four types of hand exertion. Table 5 reveals the results of all proposed models by hands. Among these models, the demographic variable of gender can be used to predict the strength, except in the palmar pinch strength for both hands. Again, measured grip strength can only be used to predict the strengths of later pinch and palmar pinch. The adjusted-*R*^2^ values were between 0.572 and 0.717, which is acceptable. On the other hand, the adjusted-*R*^2^ values were less than 0.5 for the models of two types of pressing for both hands, which means that measured grip was not a good predictor.

## 4. Discussion

### 4.1. General Linear Models Using Demographic Variables

This study tried to propose models to predict the hand strength of Taiwanese workers in the manufacturing industries according to different types of hand motions, demographic variables, and the interactions of demographic variables. When using demographic variables to predict the strength, gender, age, height, and weight were included in the models. The highest coefficient of multiple determination was 68.8% when predicting the left hand’s grip strength using gender and weight. On the other hand, the lowest coefficient of multiple determination was only 29.1% for the right hand’s ball of thumb. Gender was the most important variable in the models and it is not surprising that there was a significant difference in strength in terms of gender [11,13,17,19,20,23,29]. On the other hand, age can only be used to predict the grip strength of the right hand, and not in all models, especially for the pinch strength. This finding contradicted previous study results that indicated that age could be used to predict pinch strength [19,24]. A possible reason is the differences in the study population. In our study, all participants were field operators in manufacturing industries, not in a management department or administration. For physical activities, age may not play an important role in pinch strength. On the other hand, the study population in Mohammadia’s study and Angst’s study were from the general population, which included sedentary and retired individuals [19,24]. Height is another important variable that was used to predict strength in previous studies. However, it is surprising that height was not an important explaining factor in our models.

### 4.2. General Linear Model Using Strength of Opposite Hand and Demographic Variables

Next, we performed a multiple linear regression to predict strength using the measured strength in the opposite hand and demographic variables (Table 3). Previous studies showed that there was a high correlation between right and left hand strength in terms of the grip and pinch [11,13,14,15,17,18,19,20,26,28,29]. Our models confirm these results; the coefficient of multiple determination was greater than 75.7%. In these models, gender was still the main predictor. However, other demographic variables, e.g., age, height, and weight, were only included in 3 out of 10 models. Although the proposed models using opposite hand strength performed well, it still takes time and resources to measure the strength of all five types of hand motions. Increasing the number of trials may also result in muscle fatigue and decrease individuals’ willingness to participate in the study.

### 4.3. Prediction Models of the Hand Strength by Strength of Other Hand Motions, Demographic Variables, and Their Interactions

Therefore, we included other measurements of strength of different hand motions, demographic variables, and the interactions of demographic variables in the multiple linear regression, separately for each hand (Table 4). However, the coefficient of multiple determination for both hands slightly dropped to between 68.8 and 79% for the right hand and between 67.7% and 84.2% for the left hand. The results were acceptable. From the results, the five types of hand motions can be clearly categorized into two groups from a biomechanical standpoint. The first was the power grip and two types of pinch motions, which only require the muscles in the forearm and distal upper extremities, such as the flexor digitorum superficialis, flexor digitorum profundus, and palmaris longus. The other group includes the two types of press motions, which require whole muscle groups in the upper extremities and even the muscles in the shoulders. The measured palmar pinch strength is the main factor that can be used to predict the grip and lateral pinch strength of a specific hand. On the other hand, the measured strength of the ball of thumb can be used to predict the strength of the thumb press since the coefficient of multiple determination was higher. Regarding the demographic variables, gender is still the main variable in the models—either as a single factor or interacting with other demographic variables.

### 4.4. Prediction Models of Hand Strength by Grip Strength, Demographic Variables, and Interactions of Demographic Variables

Finally, we performed another hand-specific investigation on the predicting models, including the measured grip strength in the models. The reason is that grip strength is widely used in many disciplines, such as rehabilitation, ergonomics, gerontology, etc. However, the results were less acceptable since the coefficient of multiple determination was lower than 50% in half of the models (Table 5). Again, the results agreed with the results of the previous paragraph. Measured grip strength may not be a suitable factor in the models for pressing tasks.

In this study, the correlations between the five types of hand motions and BMI were relatively low, and we did not include BMI in the models. This is the same as the study results of Gunther et al. in 2008 [48]. Furthermore, BMI is an index of the whole body, so may not be sensitive to the localized muscles in the upper extremities.

### 4.5. Limitations and Recommendations for Future Studies

Werle et al. (2009) found that grip strength was significantly different among occupational groups, with grip and pinch strength increasing with the level of occupational demand [50]. On the other hand, Gunther et al. (2008) did not find a strength difference in the occupational group and thought that the grip difference of retired individuals should attribute to collinearity with age [48]. For our study, we only recruited volunteers from the manufacturing industry. Future studies should include people from other industries.

There are some limitations to this study. The study participants were healthy adults in the manufacturing industry and the models cannot be used to predict the strength if there are any disorders of the upper extremities, such as carpal tunnel syndrome, de Quervain’s syndrome, or trigger fingers. Second, the proposed models can only be used for individuals in Taiwan and may not be suitable for other countries or races. We plan to conduct another study to validate the proposed models.

## 5. Conclusions

This is the first study to propose models to predict the strength of five types of hand motions. Gender is the key demographic variable that can be used in the predicting models. Although power grip strength is widely used in many disciplines, it may not be a good predictor for thumb press and ball of thumb. We recommend measuring the palmar pinch strength and ball of thumb strength and using these two measurements to predict the other two types of hand strength for convenience and time saving.

## Figures and Tables

**Table 1:** Demographic and anthropometric characteristics of volunteers by sex. Values shown are mean/count (standard deviation/percent).

Sex	*N*	Age (Year)	Height (cm) ^a^	Weight (kg) ^a^	BMI (kg/m^2^) ^a^	Handspan (cm) ^a^	Hand Dominance	
							Right	Left
Male	99	39.1 (12.8)	171.9 (5.9) **	73.7 (12.8) **	24.9 (3.8) *	20.0 (1.9) **	95 (48.0%)	4 (2.0%)
Female	99	39.1 (13.1)	159.5 (5.0) **	59.0 (9.3) **	23.2 (3.4) *	17.8 (1.6) **	91 (46.0%)	8 (4.0%)
All	198	39.1 (12.9)	165.7 (8.3)	66.3 (13.4)	24.0 (3.7)	18.9 (2.0)	186 (93.9%)	12 (6.1%)

Note: ^a^: *t*-Test; * *p* < 0.05; ** *p* < 0.001.

**Table 2:** The prediction models by demographic information (gender and weight) of different types of hand strength.

Types of Hand Motions	Regression Equation	Adjusted-*R*^2^
Grip_R	62.021 − 20.092 (gender)	0.640
Grip_L	41.136 − 16.026 (gender) + 0.198 (weight)	0.688
Ball of Thumb_R	8.966 − 2.76 (gender) + 0.065 (weight)	0.291
Ball of Thumb_L	10.817 − 3.291 (gender) + 0.051 (weight)	0.297
Thumb Press_R	11.879 − 3.276 (gender)	0.322
Thumb Press_L	11.831 − 3.375 (gender)	0.349
Lateral Pinch_R	11.979 − 3.278 (gender)	0.617
Lateral Pinch_L	8.752 − 2.721 (gender) + 0.029 (weight)	0.584
Palmar Pinch_R	10.653 − 2.689 (gender)	0.449
Palmar Pinch_L	9.834 − 2.429 (gender)	0.418

Note: Age: years; gender: male = 1, female = 2; weight: kg; height: cm.

**Table 3:** Prediction models using the strength of the opposite hand and demographic information.

Type of Hand Motions	Regression Equation	Adjusted-*R*^2^
Grip_R	0.776 + 1.029 (Grip_L)	0.921
Grip_L	2.594 + 0.81 (Grip_R) − 1.987 (gender) + 0.045 (weight) + 0.045 (age)	0.929
Ball of Thumb_R	−0.196 + 0.904 (Ball of Thumb_L) + 0.015 (weight)	0.931
Ball of Thumb_L	1.979 + 0.986 (Ball of Thumb_R) − 0.57 (gender) − 0.013 (weight)	0.932
Thumb Press_R	0.401 + 0.97 (Thumb Press_L)	0.924
Thumb Press_L	0.958 + 0.915 (Thumb Press_R) − 0.376 (gender)	0.926
Lateral Pinch_R	3.304 + 0.768 (Lateral Pinch_L) − 0.871 (gender)	0.845
Lateral Pinch_L	1.502 + 0.818 (Lateral Pinch_R) − 0.454 (gender)	0.831
Palmar Pinch_R	2.837 + 0.795 (Palmar Pinch_L) − 0.759 (gender)	0.769
Palmar Pinch_L	2.012 + 0.734 (Palmar Pinch_R) − 0.454 (gender)	0.756

Note: Age: years; gender: male = 1, female = 2; weight: kg; height: cm.

**Table 4:** The best prediction models using different types of hand exertion, demographic variables, and their interaction by laterality.

Laterality	Type of Hand Motions	Regression Equation	Adjusted-*R*^2^
Right	Grip	26.676 + 2.950 (Lateral Pinch_R) − 10.420 (gender)	0.744
	Ball of Thumb	2.749 + 1.014 (Thumb Press_R) + 0.049 (weight) − 0.086 (Grip_R) − 0.008 (height) × (gender)	0.746
	Thumb Press	0.522 + 0.574 (Ball of Thumb_R) + 0.056 (Grip_R) − 0.028 (weight) + 0.177 (Lateral Pinch_R)	0.786
	Lateral Pinch	2.770 + 0.465 (Palmar Pinch_R) − 0.862 (gender) + 0.055 (Grip_R) + 0.000116 (height) × (age)	0.777
	Palmar Pinch	1.268 + 0.531 (Lateral Pinch_R) + 0.05 (Grip_R)	0.713
Left	Grip	27.996 + 2.137 (Lateral Pinch_L) − 10.181 (gender) + 1.347 (Thumb Press_L) − 0.618 (Ball of Thumb_L)	0.785
	Ball of Thumb	5.596 + 1.161 (Thumb Press_L) − 0.127 (Grip_R) − 1.952 (gender) + 0.039 (weight)	0.719
	Thumb Press	−2.949 + 0.502 (Ball of Thumb_L) + 0.078 (Grip_L) + 0.24 (Palmar Pinch_L) + 0.005 (gender) × (height)	0.782
	Lateral Pinch	1.344 + 0.711 (Palmar Pinch_L) + 0.041 (Grip_L) − 0.593 (gender) + 0.000078 (height) × (age)	0.857
	Palmar Pinch	1.313 + 0.740 (Lateral Pinch_L) + 0.072 (Thumb Press_L) − 0.000076 (height) × (age)	0.810

Note: Age: years; gender: male = 1, female = 2; weight: kg; height: cm.

**Table 5:** The prediction models using measured grip strength, demographic variables, and interaction of demographic variables by laterality.

Laterality	Type of Hand Motion	Regression Equation	Adjusted-*R*^2^
Right	Ball of Thumb	7.973 + 0.022 (Grip_R) − 0.2.381 (gender) + 0.061 (weight)	0.289
	Thumb Press	2.456 + 0.141 (Grip_R)	0.378
	Lateral Pinch	4.417 + 0.017 (Grip_R) − 1.073 (gender) + 0.000133 (height) × (age)	0.719
	Palmar Pinch	2.644 + 0.125 (Grip_R)	0.609
Left	Ball of Thumb	10.385 + 0.011 (Grip_L) − 3.123 (gender) + 0.049 (weight)	0.293
	Thumb Press	1.959 + 0.159 (Grip_L)	0.425
	Lateral Pinch	4.527 + 0.116 (Grip_L) − 0.947 (gender)	0.687
	Palmar Pinch	2.528 + 0.121 (Grip_L)	0.572

Note: Age: years; gender: male = 1, female = 2; weight: kg; height: cm.

## Data Availability

The data presented in this study are available on request from the corresponding author. The data are not publicly available due to privacy or ethical reasons.

## Abbreviations

HGS	Hand grip strength
WRULDs	Work-related upper limb musculoskeletal disorders
WHO	World Health Organization
EAWS	Ergonomic Assessment Worksheet
KIM-MHO	Key Indicator Method for Manual Handling Operations
OCRA	Occupational Repetitive Actions
CTS	Carpal tunnel syndrome
ASHT	American Society of Hand Therapists
Adjusted R2	Adjusted R-square
CV	Coefficient of variation
BMI	Body mass index
